# A Reliable and Non-destructive Method for Monitoring the Stromal pH in Isolated Chloroplasts Using a Fluorescent pH Probe

**DOI:** 10.3389/fpls.2017.02079

**Published:** 2017-12-05

**Authors:** Pai-Hsiang Su, Yen-Hsun Lai

**Affiliations:** ^1^Agricultural Biotechnology Research Center, Academia Sinica, Taipei, Taiwan; ^2^Biotechnology Center in Southern Taiwan, Academia Sinica, Tainan, Taiwan

**Keywords:** BCECF, chloroplast, fluorescent pH probe, nigericin, proton gradient, ionophore

## Abstract

The proton gradient established by the pH difference across a biological membrane is essential for many physiological processes, including ATP synthesis and ion and metabolite transport. Currently, ionophores are used to study proton gradients, and determine their importance to biological functions of interest. Because of the lack of an easy method for monitoring the proton gradient across the inner envelope membrane of chloroplasts (ΔpH_env_), whether the concentration of ionophores used can effectively abolish the ΔpH_env_ is not proven for most experiments. To overcome this hindrance, we tried to setup an easy method for real-time monitoring of the stromal pH in buffered, isolated chloroplasts by using fluorescent pH probes; using this method the ΔpH_env_ can be calculated by subtracting the buffer pH from the measured stromal pH. When three fluorescent dyes, BCECF-AM [2′,7′-bis-(2-carboxyethyl)-5-(and-6)-carboxyfluorescein acetoxymethyl ester], CFDA-SE [5(6)-Carboxyfluorescein diacetate succinimidyl ester] and SNARF-1 carboxylic acid acetate succinimidyl ester were incubated with isolated chloroplasts, BCECF-AM and CFDA-SE, but not the ester-formed SNARF-1 were taken up by chloroplasts and digested with esterase to release high levels of fluorescence. According to its relatively higher pKa value (6.98, near the physiological pH of the stroma), BCECF was chosen for further development. Due to shielding of the excitation and emission lights by chloroplast pigments, the ratiometric fluorescence of BCECF was highly dependent on the concentration of chloroplasts. By using a fixed concentration of chloroplasts, a highly correlated standard curve of pH to the BCECF ratiometric fluorescence with an *r*-square value of 0.98 was obtained, indicating the reliability of this method. Consistent with previous reports, the light-dependent formation of ΔpH_env_ can be detected ranging from 0.15 to 0.33 pH units upon illumination. The concentration of the ionophore nigericin required to collapse the ΔpH_env_ was then studied. The establishment of a non-destructive method of monitoring the stromal pH will be valuable for studying the roles of the ΔpH_env_ in chloroplast physiology.

## Introduction

Proton (H^+^) gradients across biological membranes provide the driving force for ATP synthesis and for secondary active transport of solutes between cellular compartments, which maintain different pHs for optimal biochemical reactions (Reid et al., 1966; Avron, 1977; Shingles et al., 1996; Cruz et al., 2001; Neuhaus and Trentmann, 2014; Hohner et al., 2016). The measurement of the pH of different subcellular compartments is essential for determining the proton gradient across the membranes. Many methods have been developed to trace cellular and organelle pHs, including weak-acid/base indicators, nuclear magnetic resonance, pH microelectrodes, pH-sensitive dyes, and fluorescent probes and proteins (Iles, 1981; Loiselle and Casey, 2010). All methods currently used for measuring intracellular pH have their own advantages and technical limitations. Among them, fluorescent pH probes are a cost-effective and non-destructive way to determine the pH of intracellular compartments by spectrometric or image fluorescence, and are widely used in many organisms for real-time monitoring of intracellular pH (Grant and Acosta, 1997; Scott and Allen, 1999; Alvarez-Leefmans et al., 2006; Loiselle and Casey, 2010; Sundaresan et al., 2015).

In plant cells, the optimal cytoplasm pH for physiological activities is a neutral pH of around 7.2 to 7.4; the apoplast and vacuole have acidic pH ranging from 5 to 7; the thylakoid lumen has a more dynamic and highly acidic environment that changes with light/dark cycles. The mitochondrial matrix and chloroplast stroma need to keep a relatively basic environment around pH 8 for optimization of biochemical reactions occurring in these two compartments (Shen et al., 2013; Hohner et al., 2016). Consequently, proton gradients are formed across the membranes separating different compartments, and are maintained by H^+^-transporting electron transfer chain (photosynthetic light reaction and respiratory oxidative phosphorylation) or by H^+^-pumping fueled by ATP or pyrophosphate hydrolysis (H^+^-ATPase or H^+^-pyrophosphatase) (Kurkdjian and Guern, 1989).

Methods for determining the pH of most cellular and organelle compartments are well established and are used to assess the importance of pH and proton gradients for particular biological functions. However, to date, an easy and reliable method for real-time monitoring of the chloroplast stromal pH is still lacking. It is widely accepted that the stromal pH can increase from pH 7 in the dark to pH 8 upon illumination, which is required to fully activate the enzymes for carbon fixation (Berkowitz and Wu, 1993). Upon illumination, a proton gradient of 0.12–0.36 pH units across the inner envelope membrane (ΔpH_env_) can be formed in isolated chloroplasts. The value can be determined with a destructive method called silicon oil microcentrifugation by monitoring the distribution of the radioactive weak acid [^14^C]-5-5′-dimethyloxazolidine-2,4-dione across the chloroplast envelope (Heldt et al., 1973; Robinson, 1985; Wu and Berkowitz, 1992; Heiber et al., 1995).

Additionally, ionophores such as nigericin and carbonyl cyanide m-chlorophenyl hydrazone (CCCP) can be used to collapse the proton gradients for the assessment of the importance of the proton gradients on membrane activity (Nishio and Whitmarsh, 1991). The concentrations of ionophores required to diminish a particular membrane proton gradient need to be determined empirically because different membrane systems have different lipid compositions, H^+^-translocating machinery and microenvironments to respond to the action of ionophores (Kasianowicz et al., 1984). Owing to the difficulty and technical limitations, chloroplast stromal pH is rarely determined experimentally. Instead, the levels of ionophores routinely used in other membrane systems are adopted directly without demonstrating the ionophores’ ability to completely collapse the proton gradient.

Here we report the development of an easy and reliable method for continuous and real-time monitoring of the stromal pH using a ratiometric fluorescent pH probe, BCECF [2′,7′-bis-(2-carboxyethyl)-5-(and-6)-carboxyfluorescein]. Compared to the traditional destructive silicon oil filtration technique, the advantages of our method are that it is easy, cost-effective and non-destructive, and can be used in real-time. It is, therefore, ideal for conducting continuous measurements in live organelles.

## Materials and Methods

### Reconstruction of a Fluorescence Spectrometer to Add Compatible Actinic Light

The commercial fluorescence spectrometer model FP-8300 equipped with an ETC-815 thermostat cuvette holder with stirrer and CSP-829 sample compartment lid with syringe port was purchased from Jasco International, Japan. To measure the light-dependent behavior of chloroplasts after fluorescent dye uptake, the introduction of a compatible actinic light is required. A red LED actinic light (0.5 Watts, peaking at 628 nm, emission spectrum shown in Supplementary Figure S1) was delivered 180° from the excitation beam and 90° from the emission detector. To eliminate the interference of strong actinic illumination on the excitation photons, a 550 nm Techspec Shortpass Filter (Edmund Optics, United States) was placed in front of the entrance hole of a cuvette holder. The overall light-path design, module assembly and machine validation are described in detail in the section “Results.”

### Plant Materials and Chloroplast Isolation

Pea chloroplasts were isolated from 8- to 10-day-old pea seedlings grown on vermiculite in a growth chamber at 22°C under a light intensity of 200 μmol/m^2^/s. A continuous Percoll gradient was prepared by mixing half volume of Percoll reagent (GE Healthcare) and half volume of 2× grinding (GR) buffer, and by centrifuging at 38,000 × *g* for 30 min in a fixed angle rotor (RA20A2 rotor, Himac CR22G2; Hitachi Koki, Tokyo). A 1× GR buffer containing 50 mM Hepes-KOH pH 7.3, 330 mM sorbitol, 1 mM MgCl_2_, 1 mM MnCl_2_, 2 mM EDTA, and 0.1% BSA. About 20 g of young shoots was ground in a blender at low speed for 15 s, three times in GR buffer and spun at 3,000 × *g* for 3 min, and the pelleted chloroplasts were resuspended in 2 to 3 ml of GR buffer, then centrifuged on a preformed continuous Percoll gradient for organelle fractionation at 3,900 × *g* for 13 min at 4°C (SX4250 Swinging-Bucket Rotor, Beckman Coulter, United States). The intact chloroplast fraction near the bottom of the tube was retrieved, put in a new tube and washed with GR buffer twice. Finally, isolated chloroplasts were resuspended in GR buffer at a concentration of 1 mg/ml chlorophyll, and stored on ice in the dark until use.

### Uptake and Digestion of Esterified Fluorescent pH Probes

To determine the intracellular or intra-organellar pH by fluorescent pH probes, generally, their non-fluorescent and membrane permeable esterified forms are incubated with cells or organelles. After loading into the cells or organelles, their ester bonds are digested by endogenous esterase to release fluorescent free forms that are not membrane permeable. For our experiments, three commonly used pH indicators BCECF (pKa ∼6.98), CFDA [5(6)-carboxyfluorescein diacetate; pKa ∼6.5] and SNARF-1 (seminaphthorhodafluors; pKa ∼7.5) were tested. Their uptake ability and digestibility were evaluated by feeding isolated chloroplasts of 0.5 mg/ml chlorophyll with their ester derivatives BCECF-AM (acetoxymethyl ester) (B1170, Molecular Probes), CFDA-SE (*N*-succinimidyl ester) (Cat#21888, Sigma) and SNARF-1 carboxylic acid acetate succinimidyl ester (S22801, Molecular Probes), respectively, at concentrations of 20, 80, and 80 μM, for 20 min at RT and then 10 min on ice. Thereafter, intact chloroplasts were re-isolated by a 40% Percoll cushion, washed once with GR buffer and checked by a fluorescence spectrometer (FP-8300, Jasco, Japan) or a laser confocal microscope (LSM710, Zeiss, Germany). Parameters used are described in the figure legends. To determine the sub-organellar distribution of BCECF, intact chloroplasts were lysed in hypotonic buffer containing 50 mM Hepes-KOH pH 8.0, 50 mM NaCl and 4 mM MgCl_2_ on ice for 3 min. The lysed chloroplasts were centrifuged at 3,000 × *g* for 3 min to separate the stromal fraction (supernatant) and the thylakoid lumen-containing membrane fraction (pellet). The resultant pellet was washed once with hypotonic buffer and centrifuged again. Two supernatant fractions were combined and clarified with centrifugation at 7,500 × *g* for 5 min. Two pellet fractions were resuspended in hypotonic buffer and combined. The BCECF signal was measured by the fluorescence spectrometer. An equal amount of chloroplasts that had not been incubated with BCECF was added to the stromal fraction to equalize the chlorophyll background with the pellet fraction before measurement.

### Establishment of the pH-Fluorescence Correlation Standard Curve and Measurements of Proton Gradients

Stromal pH measurements with BCECF are made by determining the ratio of emission intensity at 535 nm when the dye is excited at 490 nm (pH-dependent) vs. when the dye is excited at 440 nm (pH-independent isosbestic point). *In situ* measurements of BCECF ratiometric fluorescence was conducted in 50 mM Hepes-Tris buffer of pH 6.8, 7.2, 7.6 and 8.0 containing 330 mM sorbitol, 15 mM KCl and 1 μM nigericin (N7143, Sigma). For each measurement, the fluorescence of chloroplasts of the same concentration without BCECF was measured as a background. The ratio of the fluorescence intensity is a sigmoidal function of the [H^+^] between pH 4 and 9 with an essentially linear mid region from pH 6 to 8 (James-Kracke, 1992). To simplify the conversion of ratio-metric fluorescence intensity to the stromal pH, the standard curve was established with simple linear regression.

### Optimizing the Measurement Parameters of the Fluorimeter for Continuous Reading

Chloroplasts are very sensitive to light. Even in dim light, a photosynthetic light reaction may be triggered. The 9-Amionoacridine (9-AA) fluorescence quenching is routinely used to determine of the formation of a proton gradient across the thylakoid membrane (ΔpH_thy_) upon illumination (Evron and McCarty, 2000; Theg and Tom, 2011). Here we optimized the measurement parameters (including excitation beam width and data reading interval) of the FP-8300 fluorescence spectrometer by determining the effect of measuring light on 9-AA fluorescence quenching. One milliliter chloroplast suspension of 0.1 mg/ml chlorophyll in GR buffer containing 5 μM 9-AA and 20 μM methyl viologen (as an electron acceptor) was mixed continuously in 1-ml stirred quartz cuvette at 25°C. The 9-AA fluorescence was monitored at 455 nm emission wavelength while it was excited at 400 nm. Fluorescence of the chloroplast suspension without the addition of 9-AA was measured as background.

### Real-Time Monitoring of the Light Dependent Increase in Stromal pH and the Effect of Nigericin on Collapsing the ΔpH_env_

To verify that our method can be used for continuous and real-time monitoring of the stromal pH in live chloroplasts, the fluctuation of the stromal pH upon illumination was determined continuously. One milliliter of BCECF-loaded chloroplast suspension of 0.1 mg/ml chlorophyll in GR buffer containing 5 mM NaHCO_3_, 0.25 mM NaH_2_PO_4_, 1,000 units of catalase (as a scavenger of H_2_O_2_ produced in the light to protect chloroplasts from damage) was mixed continuously in 1-ml stirred quartz cuvette at 25°C. After a few minutes pre-equilibration, the ratiometric fluorescence of F490/440 was determined as described above. Red actinic light was delivered during 180–420 s in an 800-s time period. The fluorescence of the chloroplast suspension without BCECF was determined as background to normalize the reads. The data was collected every 5 s and the excitation shutter was opened only when the data were being collected. To test whether nigericin can collapse the light-dependent formation of the ΔpH_env_, 5 μl of 0.2 mM nigericin was injected into the illuminated chloroplast suspension to make a final concentration at 1 μM through the syringe pore of CSP-829 sample compartment lid.

## Results

### Light-Path Design for the Introduction of Actinic Light

**FIGURE 1:**
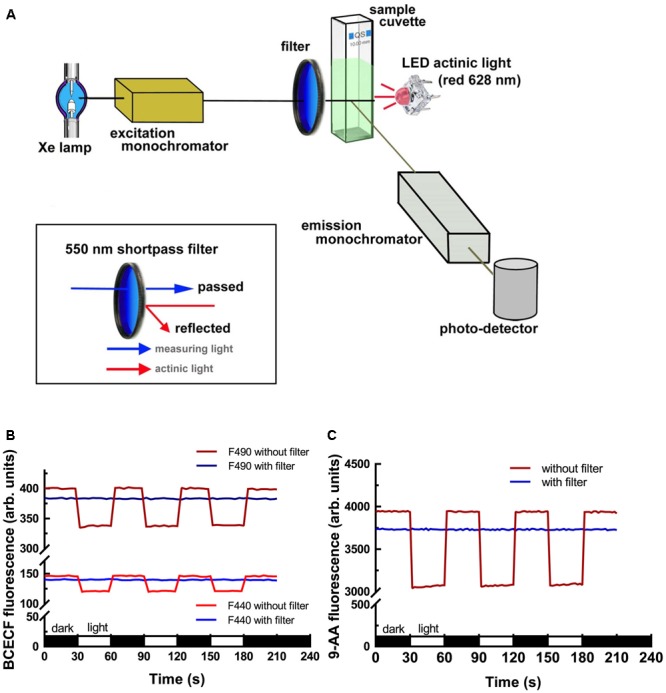
Fluorescence spectrometer modification and validation. **(A)** Illustration of the light-path and module assembly for the introduction of actinic light for measuring the light-dependent behavior of chloroplasts. Attachment of a 550 nm shortpass filter in the front of the cuvette facing the excitation beam is critical for reducing the interference of strong actinic light on excitation photons. Measurements of BCECF **(B)** and 9-AA **(C)** fluorescence showed that the use of the 550 nm shortpass filter can eliminate the interference of actinic light on the excitation photons. Without the 550 nm shortpass filter, the emitted fluorescence of BCECF and 9-AA dropped upon illumination. After adding the shortpass filter the emitted fluorescence became constant.

To measure the light-dependent pH change in isolated chloroplasts, we modified a commercial fluorescence spectrometer to add actinic light. The light-path arrangement is shown in **Figure 1A**. A LED actinic light (4 chip Piranha red LED module, peaks at 628 nm, 0.5 Watts) was placed 180° from the excitation beam and 90° from the emission detector. To eliminate the interference of strong actinic illumination on the excitation photons, a 550 nm Techspec Shortpass Filter (Edmund Optics, United States) was placed in the front of the entrance hole of the cuvette holder. Side-by-side comparison of the interference of the actinic light on the dye fluorescence with or without the shortpass filter was conducted to validate our setup. As shown in **Figures 1B,C**, the emitted fluorescence intensities of BCECF and 9-Amionoacridine (9-AA) dropped significantly upon illumination without the use of a 550 nm shortpass filter. After the attachment of the shortpass filter, the phenomenon was totally eliminated. It should be noted that there was a 4–5% reduction in the fluorescence intensity when the shortpass filter was added because the coated filter has about 95–96% transmission efficacy.

### Uptake of Fluorescent pH Probes by Isolated Chloroplasts

**FIGURE 2:**
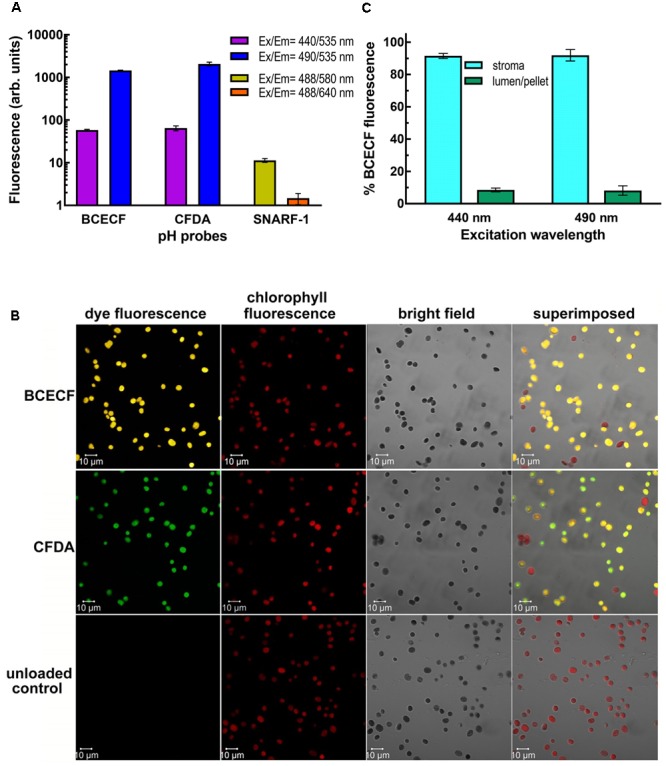
Uptake and digestion of fluorescent pH dyes by isolated chloroplasts. **(A)** Fluorescence of the dye-loaded chloroplasts of 0.1 mg/ml chlorophyll was measured by a fluorescence spectrometer. For BCECF and CFDA, emission at 535 nm was detected when the dyes were excited at 440 and 490 nm. For SNARF-1, emission at 580 and 640 nm was detected when the dye was excited at 488 nm. **(B)** BCECF- and CFDA-loaded chloroplasts were visualized with Plan-Apochromat 63× oil (NA1.4) objective by Zeiss LSM710 laser confocal microscopy. When chloroplasts were excited with Argon laser at 488 nm, the fluorescence emitted at 515–555, 510–540, and 680–797 nm was collected by Quasar spectral detector as BCECF, CFDA, and chlorophyll fluorescence signals, respectively. The percentages of BCECF and CFDA-stained chloroplasts were 84.7 ± 4.5 and 83.9 ± 4, respectively. **(C)** BCECF-loaded chloroplasts were fractionated into the stroma and the thylakoid-lumen-containing membrane pellet fractions by centrifuging hypotonically lysed chloroplasts. An equal amount of chloroplasts that had not been incubated with BCECF was added to the stroma-containing supernatant to equalize the chlorophyll background before measurements. Percentage of BCECF fluorescence at 535 nm in each fraction when excited at 440 or 490 nm is presented. Data are means of three biological repeats ± SD.

We next incubated isolated pea chloroplasts with three esterified pH-sensitive fluorescent probes, BCECF-AM, CFDA-SE, and SNARF-1 carboxylic acid acetate succinimidyl ester. After incubation for 20 min at room temperature and then 10 min on ice, the probe-loaded intact chloroplasts were re-isolated through a 40% Percoll cushion. If the non-fluorescent esterified probes could enter the chloroplasts, the probes should be digested to their fluorescent forms by the endogenous esterases. The fluorescence of the probes was monitored by the fluorimeter. Ratiometric measurements are critical to eliminate distortions of data caused by photobleaching and variations in probe loading and retention, as well as by instrumental factors such as illumination stability (O’Connor and Silver, 2007; Han and Burgess, 2010). Dual fluorescence values, one for pH-sensitive wavelengths and the other for pH-insensitive isosbestic point, have to be detected side-by-side. As shown in **Figure 2A**, BCECF-AM and CFDA-SE-fed chloroplasts produced high levels of fluorescence from the probes, indicating that the probes were taken up and digested by esterases. Only a very low level of SNARF-1 fluorescence can be detected at an intensity of about 11 and 1.5 arbitrary units at the emission wavelength at 580 and 640 nm, respectively. This result suggests that either SNARF-1 carboxylic acid acetate succinimidyl ester could not be taken up by chloroplasts or could not be digested by chloroplast esterases. We, therefore, isolated the stromal fraction from SNARF-1-incubated chloroplasts and found that a significant level of SNARF-1 fluorescence can be detected in the chlorophyll-free stromal fraction (Supplementary Figure S2). This result suggests that SNARF-1 fluorescence was concealed, possibly because of shielding of the exciting and emitted lights by pigments in the thylakoid. Particularly, its emitted light at 640 nm would be strongly re-absorbed by chlorophyll. To confirm that the fluorescent probes had been taken up by chloroplasts, fluorescent images of BCECF- and CFDA-loaded chloroplasts were visualized by laser confocal microscopy. Their fluorescence signals showed an even distribution inside the chloroplasts overlapping with the images of chlorophyll auto-fluorescence (**Figure 2B**).

Considering its relatively higher pKa value of 6.98 (near the physiological pH of stroma) and its good intracellular retention (Han and Burgess, 2010), BCECF was chosen for further development of real-time monitoring of the stromal pH. We first checked whether BCECF was also taken up into the thylakoid lumen, which would interfere with the readout on determining the stromal pH by the fluorescent probe. Fractionation of BCECF-loaded chloroplasts demonstrated that BCECF fluorescence was almost located in the stroma and was little found in the thylakoid lumen (**Figure 2C**). Sub-organellar distribution of a luminal soluble protein plastocyanin indicated that the majority of thylakoid lumen was kept intact during fractionation (Supplementary Figure S3). Taken together, lumen-resident BCECF may have little interference on pH determining, but it nearly can be ignored due to its extremely low amount.

### Establishment of a pH Standard Curve by Ratiometric Fluorescence Measurements

**FIGURE 3:**
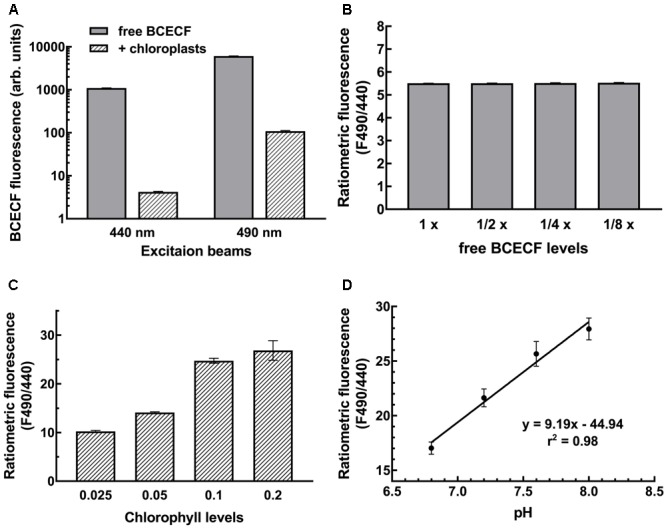
Establishment of the BCECF pH-fluorescence standard curve. **(A)** Chloroplasts attenuated the BCECF fluorescence. Fluorescence was dramatically reduced when chloroplasts were added into the BCECF-containing buffer. **(B)** A serial dilution of free BCECF in grinding buffer was made, and their ratiometric fluorescence value was determined. **(C)** Ratiometric fluorescence of BCECF-loaded chloroplasts was determined at a serial concentration of chloroplasts ranging from 0.025 to 0.2 mg/ml chlorophyll. **(D)** *In situ* measurements of BCECF ratiometric fluorescence was conducted at a fixed concentration of chloroplasts of 0.1 mg/ml chlorophyll. The pH-fluorescence standard curve was established by linear regression between pH 6.8 to 8.0.

Measurements of pH with BCECF are generally made by determining the pH-dependent emission intensity ratio (ratiometric fluorescence) detected at 535 nm when the probe is excited at 490 nm (pH-dependent) vs. the emission intensity when the probe is excited at 440 nm (pH-independent isosbestic point). *In situ* calibration is performed to first establish a standard curve representing the correlation between the ratiometric fluorescence and the pH of the samples under study. This is important because different cellular compartments have different microenvironments that will have different effects on the signal intensity. In chloroplasts, the ratiometric fluorescence is also tremendously affected by endogenous pigments of chloroplasts. Not only may the excitation beam be absorbed by chlorophylls but also the emitted fluorescence may be absorbed by chlorophyll *b* and carotenoids owing to overlapping wavelengths. As shown in **Figure 3A**, BCECF fluorescence was highly attenuated in chloroplast suspensions compared to BCECF in buffer without chloroplasts. The BCECF fluorescence in chloroplast suspension of 0.1 mg/ml chlorophyll showed about 260- and 55-fold reductions when BCECF was excited at 440 and 490 nm, respectively. This result suggests that higher interference occurred at the excitation beam at 440 nm, for which the chlorophyll has a relatively higher absorbance. To demonstrate the overall interference of chloroplast pigments on BCECF fluorescence, the full excitation spectra of BCECF affected by chloroplast suspensions were determined. It was shown that the reduction ratio of BCECF signal was highly dependent on the chlorophyll levels and the chloroplast absorption spectrum (Supplementary Figures S4A–C). Their reduction ratio was increased with the increase of the chloroplast absorbance, supporting that a relative higher reduction of BCECF signal at 440 nm is resulted from a relative higher chloroplast absorbance at 440 nm (by comparing with 490 nm). In addition, as shown in Supplementary Figure S4D, BCECF in chloroplast suspensions remained the signature of a ratiometric dye, having the pH-insensitive isosbestic point (at 440 nm) and the pH-sensitive wavelengths (usually detected at 490 nm). Without chloroplast pigment interference, the ratiometric fluorescence of BCECF changed depending on the pH, but was not affected by its concentration. When we serially diluted free BCECF in buffer from 1 to 1/8×, a constant ratiometric fluorescence (F490/F440) value of 5.5 was detected (**Figure 3B**). However, the ratiometric value of BCECF-loaded chloroplasts increased with increasing chloroplast concentration owing to the interference of chloroplast pigments (**Figure 3C**), i.e., the ratiometric fluorescence is highly dependent on the chlorophyll levels. Therefore *in situ* calibration should be conducted at a fixed concentration of chloroplast suspension.

According to this consideration, we conducted the *in situ* calibration by measuring the F490/F440 of BCECF from the chloroplast suspension. Isolated pea chloroplasts were incubated with BCECF-AM for 20 min at room temperature and then 10 min on ice, and the probe-loaded intact chloroplasts were re-isolated and resuspended to 0.1 mg/ml chlorophyll in 50 mM Hepes-Tris buffer of pH 6.8, 7.2, 7.6, or 8.0 and 330 mM sorbitol, 15 mM KCl and 1 μM nigericin. Nigericin was added to collapse all the proton gradients so the pH of chloroplasts was equal to the pH of the buffer. For each measurement, the fluorescence of chloroplasts of the same concentration without BCECF was also measured as a background. As reported previously, the ratio of the fluorescence intensity is a sigmoidal function of the [H^+^] between pH 4 and 9 with an essentially linear mid region from pH 6 to 8 (James-Kracke, 1992). To simplify the conversion of ratiometric fluorescence intensity to stromal pH, the standard curve was established with simple linear regression instead. As shown in **Figure 3D**, a coefficient of *r*-square of 0.98 was obtained, indicating a good correlation between the BCECF ratiometric fluorescence and the stromal pH and demonstrating the feasibility of our method.

### A Light-Dependent Formation of ΔpH_env_ in Isolated Chloroplasts

**FIGURE 4:**
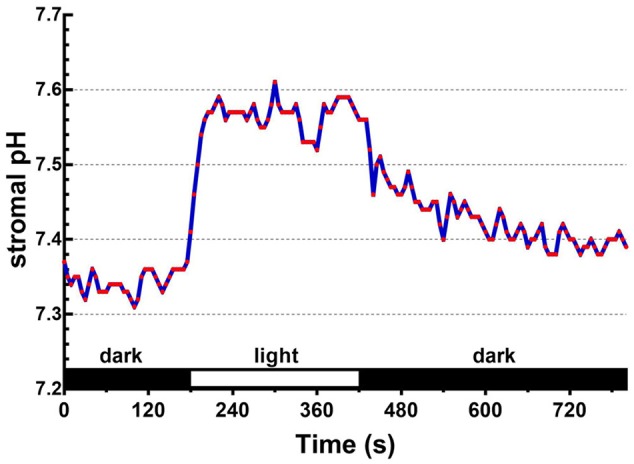
Light-dependent changes in the stromal pH can be continuously monitored. The ratiometric fluorescence of BCECF-loaded chloroplasts of 0.1 mg/ml chlorophyll was continuously measured at 5-s intervals over an 800-s period. Red actinic light was delivered between 180 and 420 s. The stromal pH was calculated through the established standard curve as shown in **Figure 3D**. Each red circle represents a datum point.

**FIGURE 5:**
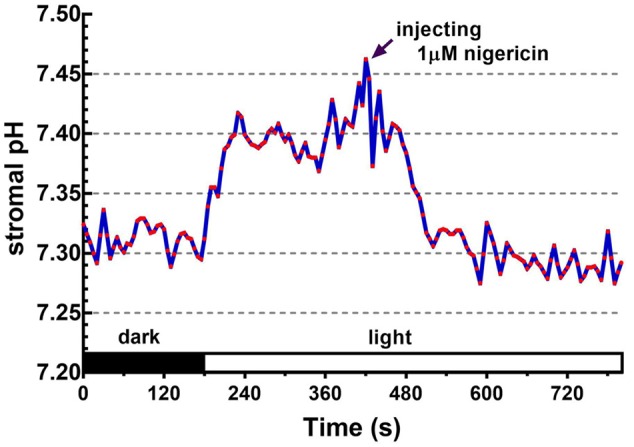
Effect of nigericin on collapsing the light-dependent formation of the ΔpH_env_. The ratiometric fluorescence of BCECF-loaded chloroplasts of 0.1 mg/ml chlorophyll was continuously measured at 5-s intervals over an 800-s period. The red actinic light was turned on at 180 s. After the formation of light-dependent ΔpH_env_, nigericin was injected into the chloroplast suspension with a final concentration of 1 μM at 420 s as indicated by the arrow. Each red circle represents a datum point.

Upon illumination, light-dependent electron transfer on the thylakoid membrane drives the movement of H^+^ from the stroma to the thylakoid lumen, which acidifies the luminal space and alkalizes the stromal compartment, and builds up not only the ΔpH_thy_ between the thylakoid lumen and the stroma, but also the ΔpH_env_ between the stroma and the cytosol. To test if our fluorescent BCECF method is capable of measuring the stromal pH in buffered isolated chloroplasts in real time, the fluctuation of the stromal pH in response to actinic light was continuously determined. A typical result of the light-dependent increase in the stromal pH is shown in **Figure 4**. The stromal pH increased sharply upon illumination, and reached a plateau in less than 1 min. The higher pH was maintained at continuous actinic light, and then declined gradually after the light was turned off. From three independent experiments, a light-dependent formation of the ΔpH_env_ can be detected reproducibly and the calculated ΔpH_env_ ranged from 0.15 to 0.33 pH units, averaging 0.25 pH units (**Table 1**), which is comparable with previous reports determined by the silicon oil microcentrifugation (see Supplementary Table S1). Furthermore, addition of 1 μM nigericin under continuous actinic light caused a decline in stromal pH to the level before the light was turned on (**Figure 5**), indicating that 1 μM nigericin under these conditions was sufficient to completely collapse the ΔpH_env_.

**Table 1:** Measured stromal pH (pH_str_) and calculated proton gradient across the inner envelope membranes (ΔpH_env_) of isolated chloroplasts from three independent experiments.

Experiment#	Dark pH_str_	Light pH_str_	pH_str_ increase upon illumination	ΔpH_env_ in the light
1	7.30	7.45	0.15	0.15
2	7.32	7.63	0.31	0.33
3	7.34	7.57	0.23	0.27
Mean	7.32	7.55	0.23	0.25
*SD*	0.02	0.09	0.08	0.09

*In all experiments, the stromal pH was determined by the ratiometric fluorescence of the BCECF-loaded chloroplasts of 0.1 mg/ml chlorophyll in a buffered medium of pH 7.3. The pH_*str*_ increase was calculated by substrating the dark pH_*str*_ from the light pH_*str*_. The ΔpH_env_ was calculated by substrating the buffered medium pH (7.3) from the light pH_*str*_.*

It should be noted that the amount of excitation light at 440 and 490 nm for exciting BCECF should be minimalized as much as possible to avoid activating the photosynthetic light reaction. According to the absorption spectra of chlorophylls, the light wavelengths at 400, 440, and 490 may have a comparable level of actinic effect on photosynthesis. The 9-AA fluorescence quenching excited at 400 nm is a sensitive way to determine the light-dependent formation of the ΔpH_thy_. We therefore performed the measurement in order to find the best balance point between good BCECF fluorescence and low photosynthetic light reaction activation. As shown in Supplementary Figure S5, a 2.5 nm bandwidth and 5-s data interval had a high level of 9-AA fluorescence while only producing 9-AA quenching of 6%. Widening the excitation beam bandwidth and shortening the reading interval resulted in quenching as high as 30%. Therefore, a good tradeoff was obtained by setting the excitation bandwidth to 2.5 nm, reading the fluorescence every 5 s for 1 s and opening the excitation shutter only when reading the data. Under these conditions there was a good balance between reducing the actinic effect and keeping a high level of fluorescence; and the actinic effect can reach an equilibrium in less than 30 s as indicated by a continuous measurement of 9-AA fluorescence quenching (Supplementary Figure S5).

## Discussion

Intracellular and organellar pHs are easily measured by fluorescent molecular probes or pH-sensitive fluorescent proteins with spectrometric or image fluorescence in many organisms (Sano et al., 2010; Shen et al., 2013). A plant organelle pH detection system based on modified pH-sensitive fluorescent proteins expressed in particular cellular compartments has been reported for Arabidopsis suspension cells (Shen et al., 2013). Unfortunately, the pH of the chloroplast stroma could not be determined because the auto-fluorescence signal of chlorophyll influenced the emission signal intensity of the pH sensor protein in light-cultured Arabidopsis PSB-L suspension cells. Plastid stromal pH could only be determined from PSB-D suspension cells incubated in the dark. It was shown that the pH value of the plastid stroma of dark-incubated PSB-D suspension cells was 7.2, which is near the cytosolic pH (around 7.3). There was almost no detectable proton gradient across the inner envelope membrane of plastids under these conditions, probably due to the fact that the plastids in the suspension-cultured cells were not fully functional because sugars were provided in the culture medium (Shen et al., 2013). These results highlight the difficulty in measuring the stromal pH of green chloroplasts due to the interference from the chlorophyll fluorescence (Yin et al., 1990; Shen et al., 2013).

Here we established an easy and reliable method for real-time monitoring of the stromal pH by the ratiometric fluorescence measurement of the pH-sensitive dye BCECF in isolated chloroplasts. The following requirements should be noted. First, because the absorption spectrum of chlorophylls overlapped with the BCECF excitation wavelengths of 440 and 490 nm, the excitation energy available to BCECF is attenuated in chloroplast suspension. Furthermore BCECF emitted fluorescence may be partially absorbed by chlorophyll *b* and carotenoids. Hence the chloroplast concentration in each sample has to be the same to equalize the interference from the chloroplast pigments (**Figure 3**). In addition, standard curves should be obtained using chloroplasts isolated from plants grown under the same conditions as the samples being studied because plants grown under different conditions would have different pigment compositions. Second, the excitation light bandwidth and shutter opening and data collection time of the fluorescence spectrometer should be set to achieve a good tradeoff between having a sufficient fluorescence signal and minimalizing the actinic effect of the excitation light on photosynthesis. In our case, narrowing the bandwidth of the excitation light to 2.5 nm, and opening the shutter only when reading the data at 5-s time interval achieved good results with the fluorescence spectrometer FP-8300 (see Supplementary Figure S5). Third, a short-pass filter in front of a sample cuvette in the path of the excitation beam is required when determining the light-dependent pH change of chloroplasts. A short-pass filter is essential to eliminate the interference of the strong actinic light on the excitation beam (**Figure 1**). With the above optimization, a BCECF fluorescence vs. stromal pH standard curve with a high correlation coefficient (*r*^2^ = 0.98) was successfully established, indicating the reliability of our method in measuring the stromal pH in buffered isolated chloroplasts. Compared to the traditional silicon oil filtration technique, our method is easy, cost-effective, and non-destructive and can be conducted in real-time. One of the most difficult parts of the silicon oil filtration technique is the determination of the accurate sizes of stromal and thylakoid luminal spaces, which is not necessary with our method.

It has long been accepted that the cytoplasm and the chloroplast stroma have a neutral pH close to 7, but upon illumination the stroma is alkalized up to pH 8 as a consequence of H^+^-pumping into the thylakoid lumen. This suggests that a proton gradient between the cytosol and the stroma is established under light (Hohner et al., 2016). By our method, the increase of stromal pH upon illumination can be continuously monitored in real-time (**Figure 4**), providing a powerful tool for studying chloroplast physiology. Establishment of the ΔpH_env_ can be routinely detected upon illumination. The value is about 0.15–0.33 pH units, which is comparable with previous reports determined by the silicon oil filtration method (Heldt et al., 1973; Wu and Berkowitz, 1992; Heiber et al., 1995). It should be noted that our non-destructive method provides more detailed information than the single-point silicon oil filtration technique. The decline in the stromal pH after turning off the actinic light was gradual and took few minutes, implying the existence of an intricate buffering system that controls the homeostasis of stromal pH. The pH decline does not seem to depend solely on the H^+^-transportation on the thylakoid membrane, since the light-dependent 9-AA quenching (representing the ΔpH_thy_) is always recovered quite fast (Köster and Heber, 1982; Theg and Tom, 2011).

## Conclusion

We have setup and validated a reliable method for the continuous, non-destructive, real-time stromal pH measurement in living chloroplasts, which will provide an important tool for future study of topics such as regulation of stromal pH changes and the importance of the ΔpH_env_.

## Author Contributions

Y-HL contributed to the isolation of intact chloroplasts and the operation of laser confocal microscopy. P-HS designed the experiments, performed fluorescence spectrometer measurements, data processing and manuscript writing.

## Conflict of Interest Statement

The authors declare that the research was conducted in the absence of any commercial or financial relationships that could be construed as a potential conflict of interest.
