# Supplementary material for: A Reliable and Non-destructive Method for Monitoring the Stromal pH in Isolated Chloroplasts Using a Fluorescent pH Probe
Source: Front Plant Sci. 2017 Dec 5;8:2079. doi: 10.3389/fpls.2017.02079 (PMC5723387; doi:10.3389/fpls.2017.02079)
Supplement: Supplementary file 1 [file Presentation_1.pdf]

Supplementary Material

A reliable and non-destructive method for monitoring the stromal pH in isolated chloroplasts using a fluorescent pH probe

Pai-Hsiang Su* and Yen-Hsun Lai

* Correspondence:
Pai-Hsiang Su
kennedy@gate.sinica.edu.tw

Table S1. Comparison of detected stromal pH (pH_{str}) and proton gradient across the inner envelope membrane (ΔpH_{env}) of isolated chloroplasts.

Reference#[*]	buffer pH	pH_{str} in the light	ΔpH_{env} in the light
1	7.60	7.85~7.92	0.25~0.32
2	7.60	7.72~7.82	0.12~0.22[**]
3	7.60	7.96	0.36
this paper	7.30	7.45~7.63	0.15~0.33

[*]1. Heldt et al (1973) Biochim Biophys Acta 314(2), 224-241.
2. Wu and Berkowitz (1992) Plant Physiol 98(2), 666-672.
3. Heiber et al (1995) Biochemistry 34(49), 15906-15917.

[**]The values were deduced from the figures of the report.

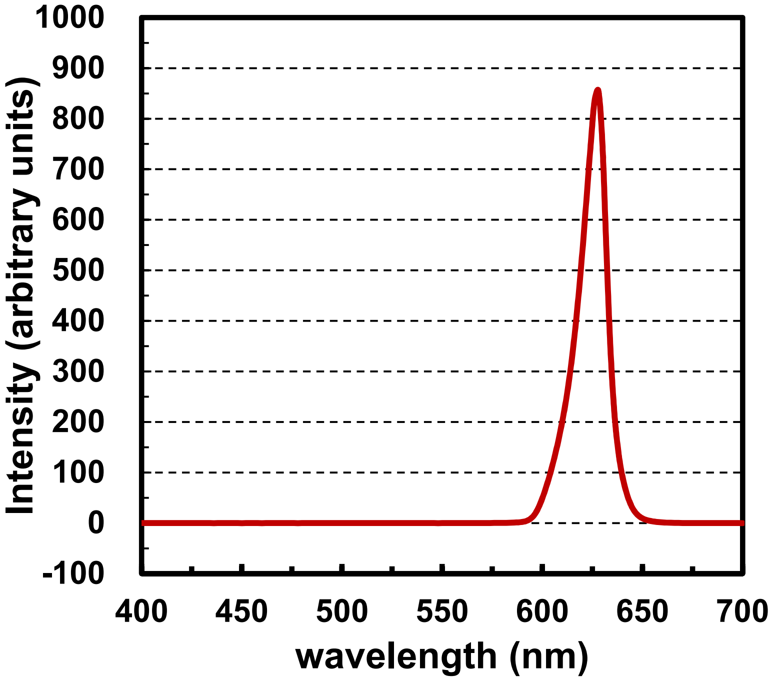

Figure S1. The light spectrum of the eagle-eyed red LED. The emission spectrum is ranged from 586 to 660 nm, and is sharply peaked at 628 nm. There isn't any detectable emission light at less than 560 nm and at more than 680 nm.

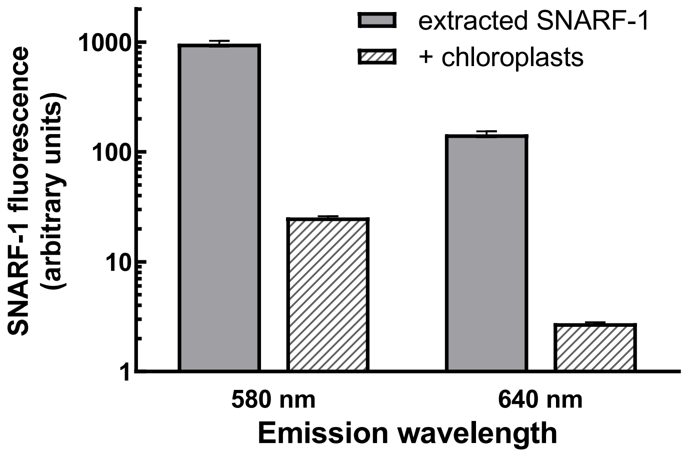

Figure S2. High levels of SNARF-1 fluorescence were detected in the extracted stroma-enriched supernatant of chloroplasts. Chloroplasts were incubated with 80 µM SNARF®-1 carboxylic acid acetate succinimidyl ester. The intact chloroplasts were re-isolated and suspended in buffer equivalent to 0.1 mg/ml chlorophyll. The loaded SNARF®-1 was extracted into the stroma-enriched supernatant by centrifuging the freeze-thaw chloroplasts at 20,000 xg for 10 min; and the chlorophyll-containing thylakoids were pelleted. High levels of SNARF®-1 fluorescence were detected at the emission wavelengths at 580 and 640 nm while exciting at 488 nm. When dye-free chloroplasts were added into the supernatant to a final concentration of 0.1 mg/ml chlorophyll (+ chloroplasts), the emission intensity had respectively about 38-fold and 53-fold reductions at 580 and 640 nm, indicating a highly shielding of exciting and emitted lights by chloroplast pigments.

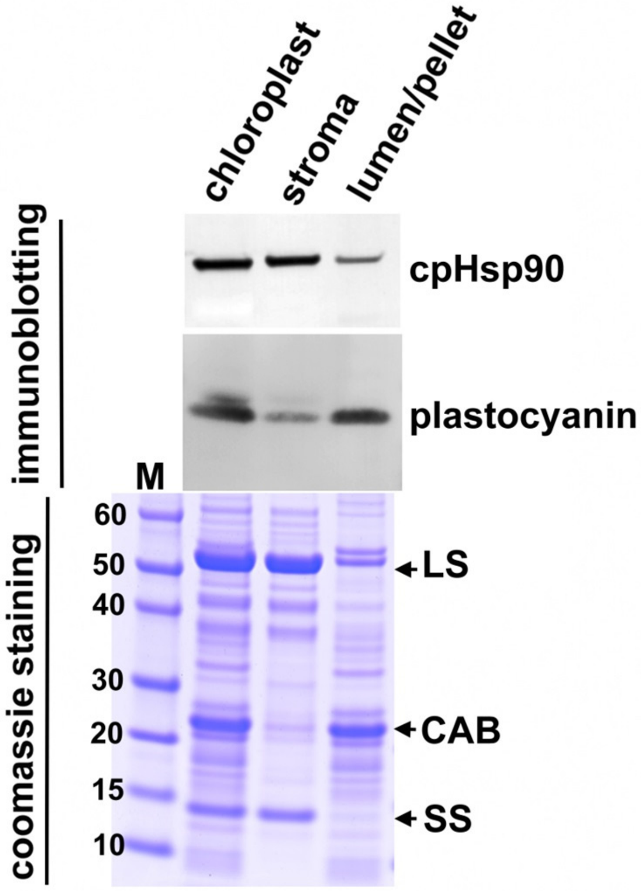

Figure S3. The majority of thylakoids remained intact after sub-organelle fractionation. To track the effectiveness of the sub-organelle fractionation of chloroplasts, each fraction equal to 1.5 µg chlorophyll (equal proportion) was loaded for NuPAGE (ThermoFisher Scientific) Bis-Tris gel electrophoresis. After resolving by PAGE, the resulting gel was visualized by Coomassie Blue staining, or blotted onto PVDF membrane for immunoblotting. The blots were decorated with antibodies against the stromal Hsp90 (cpHsp90) and luminal soluble marker plastocyanin (Cat# AS06 141, Agrisera). The majority of plastocyanin was detected in the lumen/pellet fraction, indicating the intactness of thylakoids. It is noted that a little part of thylakoid membranes was broken during fractionation based on the visualization of plastocyanin signal in the stromal fraction.

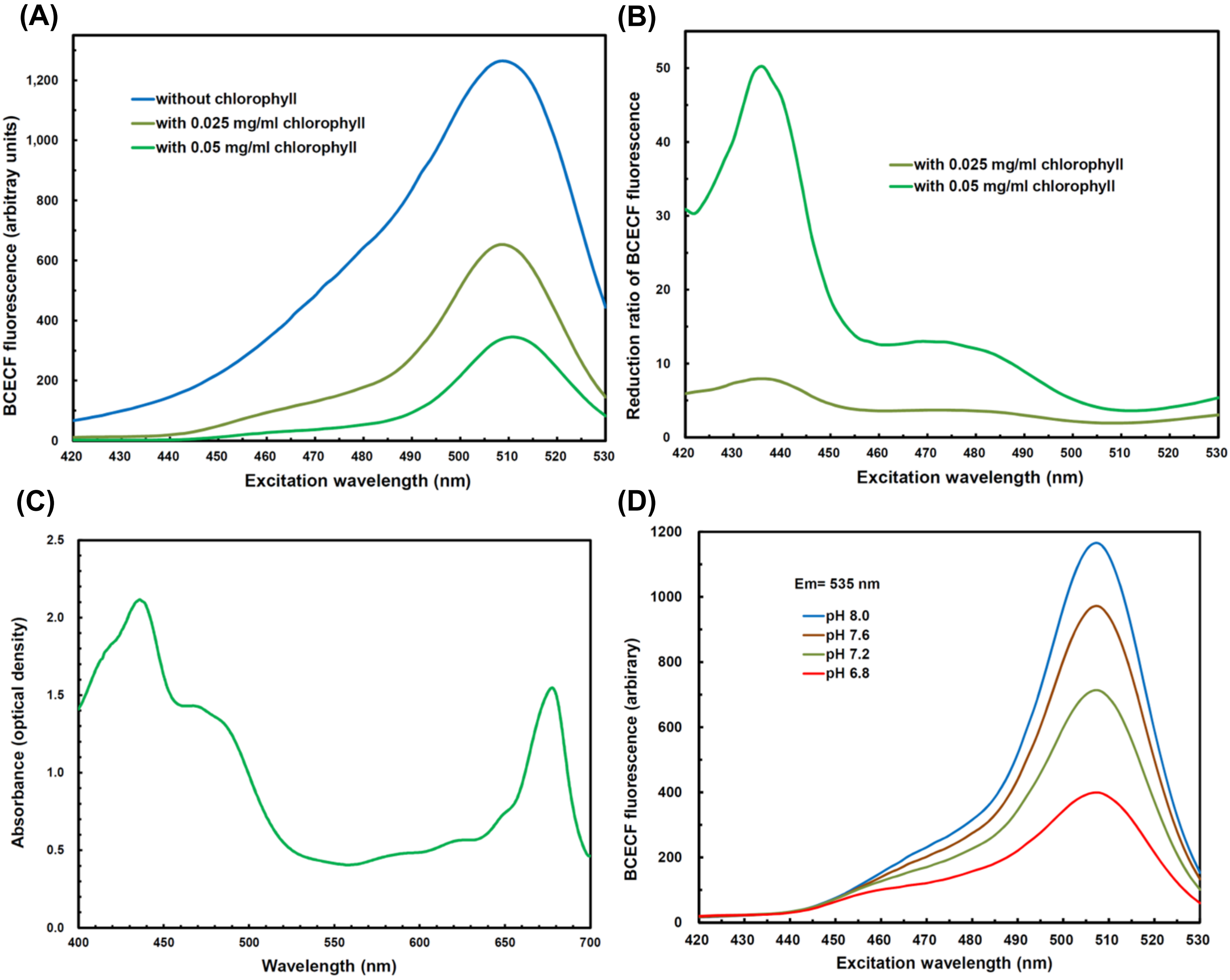

Figure S4. The interference of chloroplast pigments on BCECF fluorescence. (A) The excitation spectra of BCECF in the absence or presence of the chloroplast suspensions at indicated concentrations in grinding buffer. The emitted fluorescence at 535 nm was detected. **(B)** The reduction ratio of BCECF fluorescence derived from panel A. The values of ratio were calculated by dividing the chloroplast-free fluorescence with chloroplast-containing fluorescence. It was shown that the BCECF reduction ratio is highly dependent on the chlorophyll levels and the chloroplast absorption spectrum. The reduction ratio was increased with the increase of chloroplast absorbance. **(C)** The absorption spectrum of the chloroplast suspension of 0.025 mg/ml chlorophyll. **(D)** The excitation spectrum of BCECF in chloroplast suspensions of 0.025 mg/ml chlorophyll in different pHs of 50 mM Hepes-Tris buffer containing 15 mM KCl and 330 mM sorbitol. It was shown that BCECF in chloroplast suspensions remained the signatures of a ratiometric dye, having the pH-insensitive isosbestic point (at 440 nm) and the pH-sensitive wavelengths (usually detected at 490 nm). For above experiments, the freeze-thawed chloroplast suspensions were used.

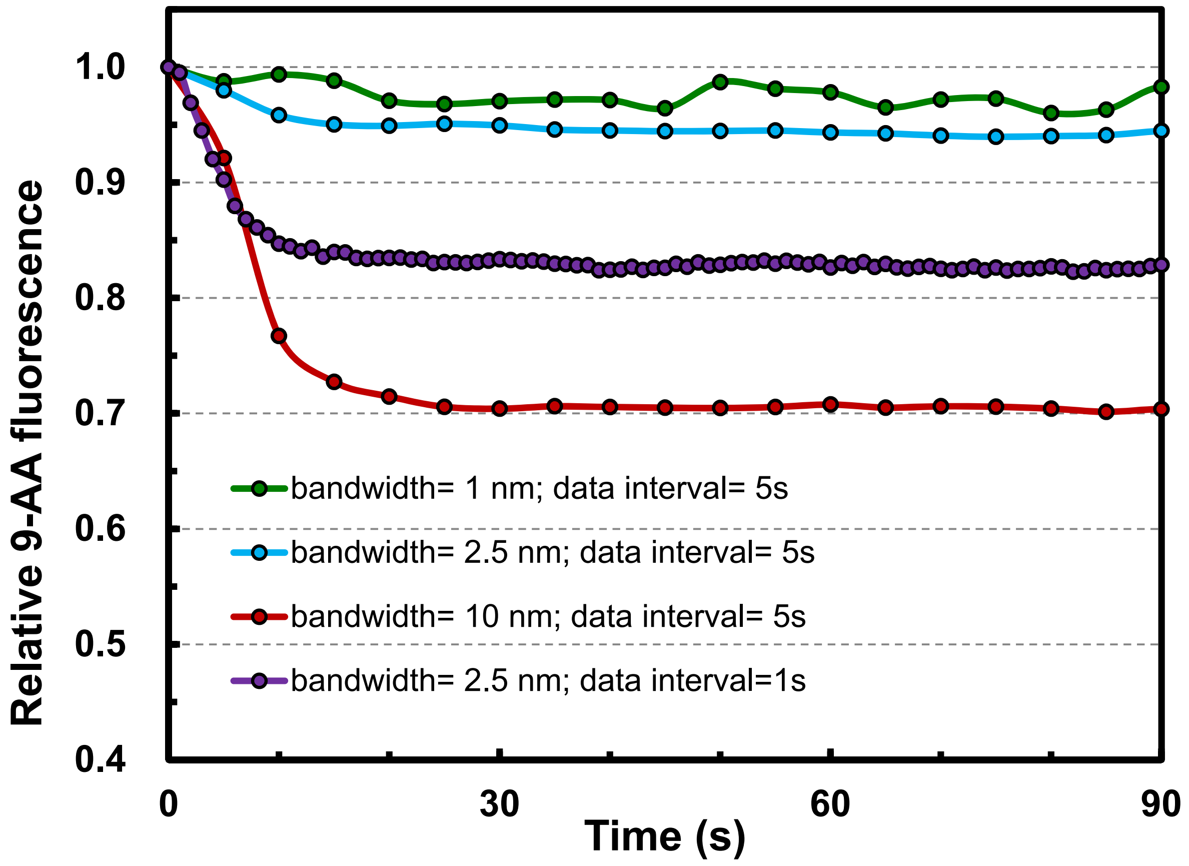

Figure S5. Optimizing the measuring parameters of fluorimeter for continuous data acquisitions. Chloroplast suspension of 0.1 mg/ml chlorophyll containing 5 μM 9-AA and 20 μM methyl viologen was mixed continuously in a stirred cuvette. Excitation beam with different bandwidth and data collection with different time interval were tested for the actinic effect of exciting light by 9-AA fluorescence quenching along the time course in the dark. For each data point, the fluorescence was scanned for 0.5 s (response time = 0.5 s). The excitation shutter was closed between acquisitions excepting the purple line, where its time interval (1 s) is too short to close/open the excitation shutter between acquisitions. The data are means of three biological repeats. Their initial 9-AA fluorescence were 55 (green line), 335 (blue line), 3650 (red line) and 327 (purple line) arbitrary units, respectively. For each condition, the fluorescence intensity of 9-AA was divided by its own initial fluorescence value to represent its relative fluorescence. It was shown that an early 9-AA fluorescence quenching, which reflects the actinic effect of exciting light, was reached an equilibrium in less than 30 s. Narrowing the excitation bandwidth, reducing the data reading frequency and opening excitation shutter only while reading were critical for minimizing the actinic effect of exciting light.
